# IgG transmitted from allergic mothers decreases allergic sensitization in breastfed offspring

**DOI:** 10.1186/1476-7961-8-9

**Published:** 2010-07-13

**Authors:** Adam P Matson, Roger S Thrall, Ektor Rafti, Elizabeth G Lingenheld, Lynn Puddington

**Affiliations:** 1 Department of Immunology, University of Connecticut Health Center, Farmington, Connecticut, USA; 2 Department of Pediatrics, Connecticut Children's Medical Center, Hartford, Connecticut, USA; 3 Department of Research, Connecticut Children's Medical Center, Hartford, Connecticut, USA

## Abstract

**Background:**

The mechanism(s) responsible for the reduced risk of allergic disease in breastfed infants are not fully understood. Using an established murine model of asthma, we demonstrated previously that resistance to allergic airway disease transmitted from allergic mothers to breastfed offspring requires maternal B cell-derived factors.

**Objective:**

The aim of this study was to investigate the role of offspring neonatal Fc receptor for IgG uptake by intestinal epithelial cells (FcRn) in this breast milk transferred protection from allergy.

**Methods:**

Allergic airway disease was induced during pregnancy in C57BL/6 female mice. These allergic mothers foster nursed naive FcRn^+/- ^or FcRn^-/- ^progeny born to FcRn^+/- ^females that were mated to C57BL/6J-FcRn^-/- ^male mice. In offspring deficient in FcRn, we expected reduced levels of systemic allergen-specific IgG_1_, a consequence of decreased absorption of maternal IgG from the lumen of the neonatal gastrointestinal tract. Using this model, we were able to investigate how breast milk IgG affected offspring responses to allergic sensitization.

**Results:**

Levels of maternal antibodies absorbed from the breast milk of allergic foster mothers were determined in weanling FcRn-sufficient or -deficient mice. Maternal transmission of allergen-specific IgG_1 _to breastfed FcRn^-/- ^offspring was at levels 10^3^-10^4 ^lower than observed in FcRn^+/- ^or FcRn^+/+ ^mice. Five weeks after weaning, when offspring were 8 wk old, mice were sensitized and challenged to evaluate their susceptibility to develop allergic airway disease. Protection, indicated by reduced parameters of disease (allergen-specific IgE in serum, eosinophilic inflammation in the airways and lung) were evident in FcRn-sufficient mice nursed as neonates by allergic mothers. In contrast, FcRn-deficient mice breastfed by the same mothers acquired limited, if any, protection from development of allergen-specific IgE and associated pathology.

**Conclusions:**

FcRn expression was a major factor in determining how breastfed offspring of allergic mothers acquired levels of systemic allergen-specific IgG_1 _sufficient to inhibit allergic sensitization in this model.

## Background

The beneficial effects of breastfeeding on infant health have been recognized for thousands of years across diverse civilizations [1]. As breast milk is the main source of passive immunity during the early months after birth, breastfeeding is considered to be the most effective means of preventing death in young children from infectious causes [2]. In addition, breastfeeding provides nutritional, developmental, psychological, social, economic, and environmental benefits [3]. While there is overwhelming evidence supporting the role of breastfeeding in protecting children from most immune-mediated diseases [4], the components in breast milk responsible for mediating this protection are not well defined.

Maternal transfer of IgG endows offspring with short-term protective immunity [5-7]. The human fetus acquires a substantial amount of maternal IgG *in utero*, transported across the placenta by the neonatal Fc receptor (FcRn) [8]. In both humans and rodents, maternal IgG is acquired from breast milk [9,10], absorbed from the gut lumen via FcRn-dependent transcytosis in intestinal epithelial cells [11-14]. It is known that mice deficient in either chain of FcRn (α-chain or β2 microglobulin) have impaired capacity to absorb maternal IgG from breast milk and accelerated decay of all IgGs, but not other Ig isotypes [13,15-19]. The structure of FcRn is well characterized [12,20] and several studies demonstrate a dynamic role of this receptor beyond the neonatal period [21,22].

It remains uncertain how maternal IgG acquired from breast milk impacts the susceptibility or severity of allergic diseases in children. It is known from animal models that offspring that receive serum fractions containing high titers of maternal antigen-specific IgG have suppressed IgE responses and enhanced IgG responses following immunization [23]. Similarly, the presence of maternal allergen-specific IgG_1 _at the time of immunization can inhibit IgE responses directed against the same allergen [24,25]. In contrast, passive transfer of allergen-specific IgG_1 _followed by local allergen challenge within the respiratory tract can induce airway eosinophilia accompanied by hyperresponsiveness to irritants (analogous to induced bronchoconstriction in asthmatics) [26]. The effect of passive immunization on exacerbation of allergic airway disease (AAD) appears mediated by enhanced allergen uptake in airway antigen presenting cells capable of activating proinflammatory CD4^+ ^T cells [27].

We demonstrated that the breast milk from allergic mothers can protect offspring from ovalbumin (OVA)-induced AAD; with the protective effect dependent on intact maternal B cell immunity [28]. Offspring nursed by wildtype allergic foster mothers have less severe OVA-induced AAD than offspring nursed by B cell deficient allergic foster mothers. The aim of the current study was to investigate the role of offspring FcRn in acquiring this maternal B cell-derived protective factor. We demonstrated that levels of OVA-specific IgG_1 _absorbed from the gut into the circulation of breastfed offspring was determined by offspring FcRn expression. Furthermore, the allergen-specific IgG_1 _absorbed from breast milk played a major role in preventing allergic sensitization in this model.

## Methods

### Animals

C57BL/6J-wildtype or -FcRn-deficient (FcRn^-/-^) mice were obtained from Jackson Laboratories (Bar Harbor, ME) or bred in our colony at the University of CT Health Center. All mice were fed sterile food and water, and housed in microisolators under specific pathogen-free conditions. Their care was in accordance with institutional and Office of Laboratory Animal Welfare guidelines.

The generation and characteristics of FcRn^-/- ^mice have been described [13]. For genotyping, tail pieces were obtained from mice prior to weaning and again at sacrifice. Genomic DNA was isolated using a Wizard Genomic DNA purification kit (Promega Corporation, Madison, WI) according to the manufacturer's instructions. PCR was performed as described [13] using FcRn o393 Forward 5'-GGATGCCACTGCCCTG-3' and FcRn o394 Reverse 5'-CGAATTCCCAGTGTATT-3'primers to amplify a 248 bp fragment from the wildtype allele. Targeting vector specific o395 Forward 5'-GGAATTCCCAGTGAAGGGC-3' and FcRn o394 Reverse were used to amplify a 378 bp fragment from the mutant allele. Gene segments were amplified using 1 μL of purified DNA in the presence of Taq DNA Polymerase (Denville Scientific Inc., Metuchen, NJ), 2.5 mM MgCl_2_, 10 mM dNTPs, and 0.4 μM o393, o394, o395 primers. After 35 amplification cycles, DNA fragments were separated by electrophoresis in a 1.5% agarose gel containing ethidium bromide and visualized under ultraviolet light. FcRn^+/+^, FcRn^+/-^, and FcRn^-/- ^mice can be distinguished using this strategy [13].

### Generation of allergic airway disease (AAD)

Mice were immunized twice, separated by 7 days, by intraperitoneal injection with 0.32 μg OVA (grade V, Sigma Chemical Co., St. Louis, MO) adsorbed to 0.08 mg Al(OH)_3 _per gram body weight. Ten to 19 days following the second immunization, animals were exposed daily to aerosolized antigen generated from 1% OVA in normal saline with a Bioaerosol Nebulizing Generator (BANG, CH Technologies, Inc., Westwood, NJ). Exposures were 1 hour for 4 or 7 consecutive days delivered via a nose-only inhalation exposure chamber with space for exposing 48 mice simultaneously (In-Tox Products, Moriarty, NM).

Allergic mothers were generated using an adaptation of this protocol essentially as described [29]. Following 7 days of primary aerosol exposure, female mice were allowed to recover for a period of 50 days and then bred with naïve C57BL/6J male mice. Pregnant mice were subjected to a secondary challenge with aerosolized OVA daily, during embryonic days (E) 11-17 of pregnancy (duration of pregnancy in C57BL/6 mice being 19-20 days).

### Sample collection for assessment of OVA-induced AAD

Severity of OVA-induced AAD was evaluated in adult mice, some of whom had been foster nursed by allergic versus naïve control mothers. Nomenclature for offspring was denoted by FcRn genotype followed by nursing mother's immune status (see Table 1). Mice were sacrificed 24 hours after the last aerosol exposure to determine serum OVA-specific Ig concentrations, distribution of airway leukocytes, and to evaluate lung histopathology. Bronchoalveolar lavage (BAL) was performed under terminal ketamine/xylazine anesthesia. Lungs from each animal were lavaged *in situ *with five-1 ml aliquots of sterile saline. Numbers of total leukocytes were obtained using a Z2™ Coulter Counter^® ^(6-20 μm; Beckman Coulter, Fullerton, CA). Differential leukocyte counts were enumerated in BAL fluid using fluorescence flow cytometry. The live leukocyte population was identified by expression of the leukocyte common antigen CD45 [30]. CD45^+ ^cells were analyzed by forward scatter (FSC) vs. CD11b to differentiate leukocyte subsets. Using this method, eosinophils (FSC low/CD11b high) were differentiated from macrophages (FSC high/CD11b intermediate) and lymphocytes (FSC low/CD11b negative). If neutrophils were present, they would be FSC intermediate-high/CD11b very high (our unpublished results).

**Table 1:** Identification of offspring based on FcRn genotype and history of exposure to the effects of maternal allergy.

Offspring mice (FcRn genotype/Nursing mother)	Prenatal Exposure (Pregnancy)	Postnatal Exposure (Nursing)
FcRn^+/- ^/B6naive^#^	B6naive	B6naive

FcRn^-/- ^/B6AAD	B6naive	*B6AAD

FcRn^+/- ^/B6AAD	B6naive	*B6AAD

FcRn^+/+ ^/B6AAD^##^	B6AAD	B6AAD

* Offspring born to naïve females were adoptively nursed by B6AAD mothers.

^# ^Positive control for disease, ^## ^positive control for protection.

For lung histology, the right lower lobe from each animal was removed, fixed with 10% formalin, processed in a standard manner, and tissue sections stained with H&E [30]. The degree of allergic lung inflammation was determined in specimens without the examiner having knowledge of the experimental condition.

### Fluorescence flow cytometry

Monoclonal antibodies used to identify airway leukocytes were anti-CD45-FITC (30-F11), -CD11b-PerCP-Cy5.5 (M1/70), -CD19-PE (1D3), -CD8α-PerCP (53-6.7), -CD4-PE (RM4-5), -CD90.2-APC (53-2.1) purchased from BD PharMingen (San Diego, CA), and -IL33 receptor-biotin (T1/ST2, [31]) purchased from MD Biosciences (St. Paul, Minnesota). Cy5-conjugated streptavidin purchased from Jackson Immuno Research (West Grove, PA) was used to identify cells labeled with biotinylated antibodies. Cells (10^4^-10^6^) were incubated with 100 μl of appropriately diluted antibodies in PBS containing 0.2% BSA and 0.1% NaN_3 _for 30 min at 4°C, and then washed with the same buffer. H-2K^b ^tetramer containing the OVA-derived peptide SIINFEKL was generously provided by Dr. Leo Lefrançois (University of CT Health Center, Farmington, CT) and labeling of OVA-specific CD8^+ ^cells was as described [32]. Relative fluorescence intensities were determined on a 4-decade log scale by flow cytometric analysis using a FACSCalibur™ (Becton Dickinson, San Jose, CA).

### Determination of serum IL-5 and OVA-specific Ig levels

In some experiments, serum was collected 24 hours after the first aerosol challenge for measurement of IL-5 levels [28]. Serum IL-5 concentrations were determined by ELISA (Pierce Biotechnology Inc., Rockford, IL). The assay was performed according to the manufacturer's recommendation. The minimum concentration of IL-5 detectable with this assay is 1.0 pg/ml.

Serum OVA-specific Ig levels were measured by ELISA using isotype-specific capture antibodies. BD Falcon Microtest™ plates (BD Falcon, Franklin Lakes, NJ) were coated with rat anti-mouse IgG_1 _(A85-3), IgE (R35-72) (BD PharMingen) or goat anti-mouse IgA (Southern Biotechnology Associates), at 2 μg/ml in 0.1 M Carbonate (pH 9.5) for 16 hours at 4°C. After blocking non-specific binding, isotype-specific antibodies were captured in duplicate, as 3-4, two-fold serial dilutions of serum (within established linear ranges of the standard for each individual isotype). Detection of antigen-specific antibodies was with OVA-digoxigenin conjugates followed by anti-digoxigenin-peroxidase (Roche Diagnostics, Indianapolis, IN) [29,33]. Development was with the TMB microwell peroxidase substrate system (Kirkegaard & Perry Laboratories, Gaithersburg, MD) and A_450 _measured with a Biorad Model 480 microplate reader (Hercules, CA). Limits of detection for OVA-specific IgG_1_, IgA, and IgE antibodies in the ELISA were 0.3 ng/ml, 10 ng/ml, or 5 ng/ml. Limits of detection in serum samples were determined by the dilution required to achieve positive readings relative to the lowest reproducible standard concentration, thus were 30, 1000, or 50 ng/ml, respectively.

### Statistical analysis

Results are expressed as mean ± standard error of the mean (SEM). Differences in antibody levels, airway inflammatory cells, and cytokine levels between groups were determined using nonparametric Mann-Whitney or Kruskal-Wallis tests. All statistical comparisons were performed with Prism 4 (GraphPad Software, San Diego, CA). Statistical significance was defined as a p value ≤ 0.05. Half life was calculated using the following formula: *t*_1/2 _= (log 0.5/(log A_e_/A_0_)) × *t*, where *t*_1/2 _is the half-life of antibody decay, A_e _is the amount of antibody remaining, A_0 _is original amount of antibody at day 0, and *t *is elapsed time [34].

## Results

### FcRn-deficient mice were susceptible to OVA-induced AAD

**Figure 1:**
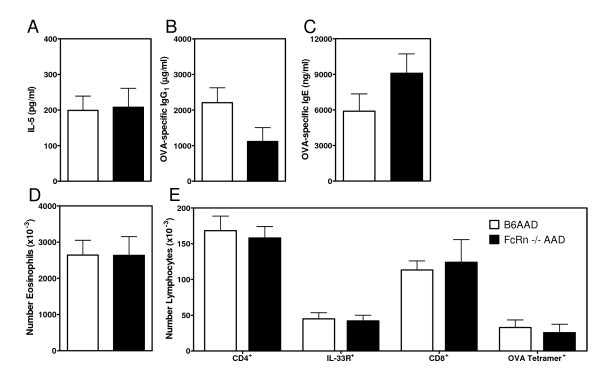
**Similar parameters of OVA-induced AAD in wildtype or FcRn deficient mice**. Five to 6 week old female C57BL/6J wildtype (B6AAD) or FcRn deficient (FcRn^-/- ^AAD) mice were given two immunizations with OVA-Al(OH)_3 _followed by challenge for 7 days with 1% aerosolized OVA (daily exposure time 60 min). Parameters of disease severity measured were **(A) **serum IL-5 concentrations determined 24 hours after the first aerosol exposure; **(B and C) **serum OVA-specific Ig titers determined 24 hours after the last aerosol exposure; and **(D) **distribution of airway leukocytes determined by fluorescence flow cytometry as described in the *Methods*. Numbers of IL-33R^+ ^or OVA-tetramer^+ ^cells were of CD4^+ ^and CD8^+ ^T lymphocytes, respectively. Results expressed as means ± SEM and represent 5-6 mice per group. There were no statistical differences in disease parameters between groups. Similar parameters of disease were obtained in an independent experiment.

Prior to performing adoptive nursing studies to elucidate the role of ingested maternal allergen-specific IgG_1 _in protecting offspring from AAD, it was necessary to determine whether wildtype and FcRn^-/- ^mice developed comparable parameters of allergic disease. Five to 6 week old C57BL/6J wildtype (B6) or FcRn^-/- ^female mice were immunized with OVA adsorbed to Al(OH)_3 _and challenged with aerosolized OVA as described in the *Methods*. Serum collected 24 hours after the first aerosol challenge demonstrated equivalent levels of IL-5 in B6AAD and FcRn^-/-^AAD mice (Figure 1A). Following sensitization, prior to aerosol challenge, serum IL-5 in immune competent mice is minimal [28]. Similarly, during acute disease following 7 days of OVA aerosol challenge, comparable levels of OVA-specific IgG_1 _and IgE were present in the serum from B6AAD and FcRn^-/-^AAD mice (Figure 1B and 1C). Airway leukocyte populations recovered from the BAL were virtually identical with equivalent numbers of eosinophils, lymphocytes (Figure 1D and 1E), and macrophages (10-100 × 10^3 ^cells per mouse, data not shown) represented. In addition, similar numbers of T lymphocytes potentially participating in disease pathogenesis were recovered from the airways of B6AAD or FcRn^-/-^AAD mice. These T cell subsets included Th2 cells (IL-33R^+^CD4^+^) [31] and OVA-specific CD8^+ ^cells (OVA Tetramer^+^CD8^+^) (Figure 1E). Routine histology of lung sections obtained from B6AAD or FcRn^-/-^AAD mice demonstrated patterns of perivascular and peribronchial inflammation, predominantly composed of lymphocytes and eosinophils, typical of the pathology we consistently observe in this model (data not shown) [28,35]. These data demonstrated that FcRn played little or no role in development of allergen-specific T and B cell responses and eosinophilic inflammation of the lungs and airways when mice were subjected to this acute model of OVA-induced AAD. Similar airway eosinophilia and histological features between FcRn-sufficient and -deficient mice using a model of mild AAD were recently reported by Nakata et al. [36].

### Adoptive nursing strategy

**Figure 2:**
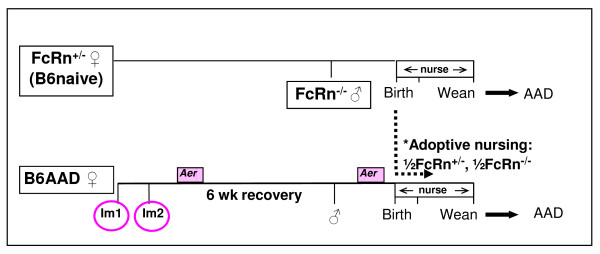
**Strategy to determine the role of "offspring" FcRn in the maternal transmission of allergic protection**.

To determine the contribution of transferred maternal allergen-specific IgG_1 _in the ability of breast milk from allergic mothers to protect offspring from AAD, we performed the experiment outlined in Figure 2. Naive C57BL/6J-FcRn^+/- ^females (B6naive) were mated to C57BL/6J-FcRn^-/- ^males, generating FcRn^+/- ^or FcRn^-/- ^progeny. Within 24 hours of delivery, pups with or without FcRn were adoptively nursed by B6AAD foster mothers. Using this strategy where all fostered pups were born to naïve mothers, acquisition of maternal allergen-specific Igs was restricted to breast milk. In this experiment, FcRn^-/- ^offspring were expected to have reduced systemic levels of OVA-specific IgG_1 _as a consequence of decreased absorption of maternal IgG from the lumen of the neonatal gastrointestinal tract [13]. Five weeks following weaning, all offspring were subjected to allergic sensitization and aerosol challenge to induce AAD as described in the *Methods*. Requisite controls to evaluate how acquiring allergen-specific IgG_1 _in breast milk affected severity of AAD were FcRn^+/- ^pups that were born to and remained with their naïve FcRn^+/- ^mothers (positive controls for disease) and wildtype FcRn^+/+ ^pups that were born and remained with their B6AAD mothers (positive controls for protection) [28].

### Levels of OVA-specific IgG_1 _absorbed from breast milk of allergic mothers

**Figure 3:**
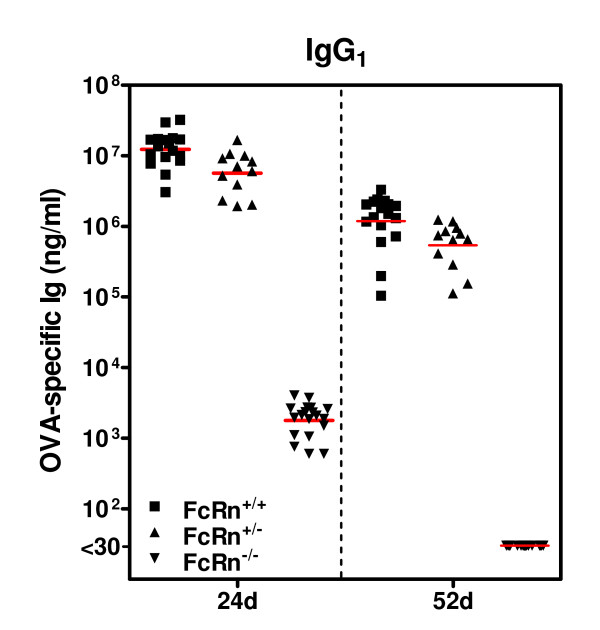
**Absorption of OVA-specific IgG**_**1 **_**by breastfed offspring was determined by offspring FcRn expression**. Naive C57BL/6J-FcRn^+/- ^females (B6naive) were mated to C57BL/6J-FcRn^-/- ^males. Progeny of this mating were FcRn^+/- ^or FcRn^-/-^. C57BL/6J OVA-induced AAD (B6AAD) foster mothers were generated (as described in the *Methods*) and within 24 hours of delivery, pups with or without FcRn were adoptively nursed by B6AAD foster mothers. Serum was collected from FcRn^+/+^, FcRn^+/-^, or FcRn^-/- ^offspring at weaning (24 days of life) and 52 days of life (1 week prior to OVA-immunization) and concentrations of OVA-specific Igs were measured by ELISA. OVA-specific Igs were absent from the serum of pups nursed by B6naive mothers (data not shown). Results are presented as 12-19 individual mice per group and the red line is the mean. There were no significant differences in serum concentrations of OVA-specific IgG_1 _antibodies between FcRn^+/+ ^and FcRn^+/- ^offspring at 24 days or 52 days of life. At 24 days of life, serum OVA-specific IgG_1 _concentrations were significantly lower in FcRn^-/- ^offspring when compared to FcRn^+/+ ^or FcRn^+/- ^offspring (p ≤ 0.01). At 52 days of life, OVA-specific IgG_1 _antibodies were no longer detected in the serum of FcRn^-/- ^offspring (limit of detection 30 ng/ml).

FcRn^+/- ^or FcRn^-/- ^offspring were nursed by B6AAD foster mothers using the adoptive nursing strategy (Figure 2), FcRn^+/+ ^offspring were nursed by their own B6AAD birth mothers. Sera were obtained from FcRn^+/+^, FcRn^+/-^, or FcRn^-/- ^offspring immediately prior and 4 weeks after weaning (at 24 and 52 days of life) for measurement of passively acquired maternal antibodies. As anticipated, at 24 days of life FcRn^+/+ ^and FcRn^+/- ^offspring had similar OVA-specific IgG_1 _serum concentrations (14,280 ± 1861 μg/ml and 6,954 ± 1259 μg/ml respectively; Figure 3). In contrast, FcRn^-/- ^offspring displayed significantly reduced OVA-specific IgG_1 _serum concentrations (< 6 μg/ml). Thus, at weaning OVA-specific IgG_1 _antibodies were evident in the serum of FcRn^-/- ^offspring nursed by B6AAD mothers, however the magnitude was 10^3^-10^4 ^lower than that observed in FcRn^+/+ ^and FcRn^+/- ^offspring. No OVA-specific antibodies were detected in the serum of pups nursed by B6naive mothers (data not shown).

At 52 days of life (4 weeks after weaning and 1 week prior to the 1^st ^OVA immunization), OVA-specific IgG_1 _concentrations were approximately 10 fold lower in FcRn^+/- ^and FcRn^+/+ ^offspring than detected at 24 days of life (weaning). At this time, OVA-specific IgG_1 _antibodies were no longer detected in the serum of FcRn^-/- ^offspring (limit of detectection was 30 ng/ml based on serum dilution of 1:100). There was no difference in the t_1/2 _of ingested maternal IgG_1 _in serum of FcRn^+/+ ^or FcRn^+/- ^offspring (~8.5 days) when calculated from OVA-specific IgG_1 _levels in individual mice at 24 days (weaning) and 52 days of life. The t_1/2 _values were similar to those previously reported (9 days) when adult FcRn^+/- ^mice were injected intraperitoneally with tracer anti-TNP IgG_1 _[13]. Given the inability to detect OVA-specific IgG_1 _antibodies at 52 days of life in FcRn^-/- ^offspring, we were unable to calculate the t_1/2 _of maternal IgG_1 _in these mice. Previous studies have established that IgG decay is accelerated in mice lacking FcRn [13,15,16]. Using the reported t_1/2 _of 1.4 days for IgG decay in serum of FcRn^-/- ^mice [13], the amount of maternal OVA-specific IgG_1 _in FcRn^-/- ^offspring at 52 days of life was calculated to be 1.9 × 10^-3 ^ng/ml, which is well below the limit of detection (30 ng/ml) in the ELISA assay. At 52 days of life, OVA-specific IgA or IgE antibodies were not detected in the serum of FcRn^+/+^, FcRn^+/-^, or FcRn^-/- ^offspring nursed by B6AAD mothers (unpublished results; limits of detection: 1000 ng/ml and 50 ng/ml respectively based on serum dilutions). However, using the levels of maternal OVA-specific IgA present in offspring serum at weaning and the reported t_1/2 _of injected IgA of 1.4 days [13], the maximum level of maternal OVA-specific IgA present at the time of the first immunization would be < 0.2 pg/ml.

### Maternal allergen-specific IgG_1 _prevented allergic sensitization

**Figure 4:**
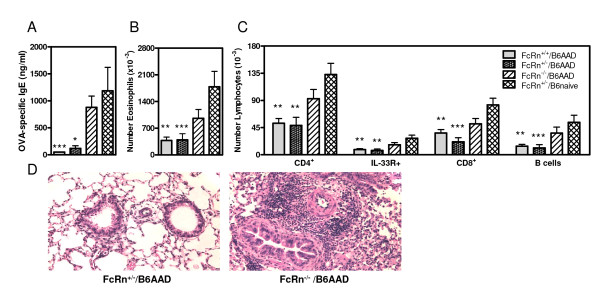
**FcRn expression was required for offspring to obtain the protective factor(s) from breast milk**. Pups are those identified in Table 1. Following weaning, allergic airway disease was elicited in 8 week old offspring by two immunizations with OVA-Al(OH)_3 _followed by challenge for 4 days with 1% aerosolized OVA (daily exposure time 60 min). Parameters of disease severity measured were **(A) **OVA-specific IgE titers determined by ELISA; and **(B and C) **distribution of leukocyte populations in the airways determined by fluorescence flow cytometry. **(D) **Representative lung sections after OVA-aerosol challenge. Results expressed as means ± SEM and represent 12-19 mice per group. * p ≤ 0.05, ** p ≤ 0.01, or *** p ≤ 0.001 when compared to FcRn^+/-^/B6naive offspring (positive controls for disease) similarly subjected to AAD. Similar differences in OVA-specific IgE titers and histopathology between groups were obtained in an independent experiment.

Adult (59 day old) mice (identified as described in Table 1) nursed until 24 days of age by naïve or allergic mothers were subjected to OVA-induced AAD as described in the *Methods*. As expected, after immunization and 4 day aerosol challenge, a robust AAD response was observed in FcRn^+/- ^offspring nursed by B6naive mothers (FcRn^+/-^/B6naive, positive controls for disease). This was demonstrated by elevated serum OVA-specific IgE levels (1,187 ng/ml ± 435 ng/ml; Figure 4A) airway eosinophils (1,786 ± 406 × 10^3^; Figure 4B), mononuclear cells (381 ± 54 × 10^3^, data not shown), and lymphocyte subsets (Figure 4C). In contrast, FcRn-sufficient offspring nursed by B6AAD mothers (FcRn^+/+^/B6AAD [positive controls for protection] and FcRn^+/-^/B6AAD) demonstrated attenuated parameters of disease as compared to those observed in the positive control FcRn^+/-^/B6naive mice. Most notable and reproducible between experiments was the greater than 10-fold lower levels of OVA-specific IgE observed in the serum of FcRn^+/+^/B6AAD (<50 ng/ml; Figure 4A) and FcRn^+/-^/B6AAD (120 ng/ml ± 48 ng/ml; Figure 4A) offspring. Ten-fold lower levels of OVA-specific IgG_1 _were also observed in serum from the same groups of mice (data not shown). Similarly, a 4-fold reduction in numbers of eosinophils (Figure 4B), 2- to 3-fold reduction in numbers of mononuclear cells (data not shown), and decreased numbers of lymphocyte subsets (Figure 4C) were recovered from the airways of FcRn^+/+^/B6AAD and FcRn^+/-^/B6AAD offspring as compared to the FcRn^+/-^/B6naive positive controls. This included a 3-fold reduction in the number of airway Th2 cells (IL33R^+ ^CD4^+^) (Figure 4C).

Interestingly, FcRn-deficient offspring nursed by B6AAD mothers (FcRn^-/-^/B6AAD) were not as protected from developing OVA-induced AAD as FcRn-sufficient offspring that were nursed by the same B6AAD mothers. Similar concentrations of OVA-specific IgE were observed in the serum of FcRn^-/-^/B6AAD offspring (924 ng/ml ± 214 ng/ml) as compared to FcRn^+/-^/B6naïve controls. Analysis of BAL leukocytes also demonstrated similar numbers of airway eosinophils (958 ± 233 × 10^3^; Figure 4B), mononuclear cells (266 ± 41 × 10^3^, data not shown), and lymphocyte subsets (Figure 4C). Furthermore, histopathologic examination of lung tissue obtained from FcRn^-/-^/B6AAD offspring demonstrated extensive perivascular and peribronchiolar cuffing, and eosinophilic inflammation, similar to that previously reported for wildtype C57BL/6 mice in this model (Figure 4D) [35]. In contrast, there was notably less allergic inflammation in lung tissue obtained from FcRn^+/+^/B6AAD and FcRn^+/-^/B6AAD offspring (Figure 4D). Thus, consistent with our previous study [28], FcRn-sufficient offspring nursed by wildtype OVA-immune mothers were protected from developing severe OVA-induced AAD. In contrast, limited, if any, protection from development of OVA-induced AAD was transmitted from allergic mothers to FcRn-deficient offspring.

## Discussion

There is overwhelming evidence supporting the role of breastfeeding in protecting children from most immune-mediated diseases [4]. Despite this, it is not clear whether this applies to prevention of allergic disease in situations when mothers are allergic. Possible explanations for the inconsistent effects of breastfeeding on allergy and asthma prevention may be the immunologic complexities of breast milk itself and potential changes in composition in the context of maternal allergy or allergen exposure. Breast milk contains a multitude of biologically active components and some elements are thought to protect the infant from developing allergies, whereas others might promote allergic sensitization [37].

We recently demonstrated that transmission of resistance to AAD from allergic mothers to nursing offspring is dependent on B cell-derived factors in breast milk [28]. By comparing offspring of mothers with OVA- versus BSA-induced lung disease, we also established that the maternally transferred protection from AAD is antigen-specific [29]. Based on these findings, we hypothesized that antigen-specific Igs in breast milk were major contributors to this protective effect. We previously demonstrated that allergen-specific IgG_1_, IgA and IgE are absorbed from the neonatal gastrointestinal tract into the systemic circulation of naïve mice nursed by allergic mothers [28,29]. No allergen-specific IgG_2a _is elicited following immunization with OVA adsorbed to Al(OH)_3 _or after the aerosol challenge in our model of AAD, thus is not absorbed by offspring nursed by allergic mothers [29]. In the present study, although maternal allergen-specific IgG_1_, IgA and IgE were present at weaning in naïve FcRn-sufficient mice foster nursed by allergic mothers, it appeared that allergen-specific IgG_1 _was the only isotype whose levels were sustained until allergic sensitization. This was not the case in FcRn-deficient mice where the low levels of maternal allergen-specific IgG_1 _present at weaning were undetectable at the time of immunization. In mice, as in humans, the transfer of breast milk IgG across the intestinal epithelium is mediated by FcRn [9]. Based on these and other data presented in this report, we were able to show that expression of FcRn was important for offspring to acquire sufficient levels of allergen-specific IgG_1 _from the breast milk of allergic mothers to prevent allergen-specific IgE responses. A distinct experimental strategy to address this research question was recently reported by Nakata et al. [36]. Importantly, the data derived from their studies led them to the same overall conclusion, that maternal IgG affects development of allergy in offspring. Thus, the two studies synergize to advance the understanding of the biology of FcRn as it applies to uptake of maternal IgG from the lumen of the gastrointestinal tract, and of how absorbed maternal allergen-specific IgG and offspring FcRn contribute to enhancing protection from allergic sensitization and disease.

To determine the role of FcRn in the postnatal acquisition of allergic protection, it was necessary to establish that FcRn-deficient mice were competent to develop OVA-induced AAD. After OVA-immunization and aerosol challenge, FcRn^-/- ^AAD mice demonstrated equivalent parameters of acute disease as wildtype B6AAD mice (this report and [36]). Of particular interest were similar titers of OVA-specific IgG_1 _antibodies in FcRn^-/- ^AAD and B6AAD mice in serum collected 24 hours after the last aerosol exposure. Thus, despite the accelerated decay of IgG in FcRn-deficient mice [13,15,16], the initial antibody titers following aerosol challenge were unaffected. These results suggest that lymphocyte responses to allergic sensitization and challenge, including the generation of OVA-specific memory CD4^+ ^T cells and B cells, were intact in FcRn-deficient mice. Furthermore, differentiation of memory B cells to antibody-producing plasma cells appeared unaffected by the absence of FcRn.

FcRn^-/- ^offspring had impaired capacity to absorb OVA-specific IgG_1 _from the breast milk of allergic mothers. At 24 days of life, 10^3 ^- 10^4 ^lower levels of antigen-specific IgG_1 _were detected in the serum of FcRn^-/- ^offspring as compared to FcRn^+/+ ^or FcRn^+/- ^offspring nursed under the same conditions. This is consistent with what is known regarding the significant role of FcRn in mediating absorption of breast milk IgG [13,17]. However, in the previous study, while the TNP-specific IgG_1 _injected into pregnant mice was present in the serum of breastfed FcRn^+/- ^neonates (10-20 μg/ml), it was not detected (<80 ng/ml) in their littermate FcRn^-/- ^mice [13]. Thus, the existence or impact of an FcRn-independent component of maternal IgG uptake has not been appreciated. It is possible the low levels of antigen-specific IgG_1 _detected in the serum of FcRn^-/- ^offspring are acquired via passive diffusion across the intestinal epithelium, although it remains to be determined whether this is the case. It is known that this mucosal barrier is more permeable in neonates with gut "closure" (cessation of Ig absorption) occurring at weaning [10].

Although we demonstrated that FcRn-independent uptake of maternal IgG can occur in neonatal mice, we found that >99.9% of IgG absorbed in wildtype mice was via an FcRn-dependent mechanism (see Figure 3). In addition to mediating transcytosis of IgG across the intestinal epithelium [11-13], FcRn modulates IgG homeostasis [13,15,16]. Thus, we were able to perform the first study that quantified the rate of decay of absorbed maternal IgG_1 _acquired exclusively from breast milk (~8.5 days). As compared to IgG acquired via intravenous injection, it is possible that ingested IgG selected by FcRn for absorption from the gut lumen has a higher binding affinity for FcRn systemically, and thereby has increased protection from catabolism. It is not clear whether or not this is the case since a direct comparison of half-lives of the same population of IgG_1 _molecules following ingestion or injection of mice at the same age has not been made. Furthermore, there appears to be a lack of consensus in the field regarding the t_1/2 _of injected IgG [13,15,16,18,19]. The explanation for the diversity in results from different laboratories is not obvious, but could be due to different routes of injection - intraperitoneal versus intravenous, or structural features of the injected monoclonal antibodies tested that contribute to their inherent strengths of interaction with FcRn.

Other studies demonstrate that under the appropriate experimental conditions, breast milk may be protective against [28,36,38-41] or increase susceptibility to [42] the development of allergic disease in offspring. It is important to understand the mechanistic basis for differences in the effect of breast milk during this early period of immune maturation. In our studies, it is possible that maternal antigen-specific IgG_1 _absorbed into the systemic circulation of offspring, neutralized the antigen - clearing it from the circulation prior to its recognition by cells of the adaptive immune system. This is supported by data from offspring nursed by allergic mothers where few, if any, FcRn-sufficient offspring produced allergen-specific IgE at 7 days after the second intraperitoneal immunization (data not shown). Neutralization of pathogens is known to occur when infants receive certain live vaccines (e.g. measles virus) in the presence of preexisting maternal antibodies. This is a major factor for delaying infant immunization until the majority of maternal antibodies have disappeared [43,44]. The functions of maternal antibodies in determining immune parameters in offspring can be influenced by the presence or absence of antigen and the ratio between them [45]. Interestingly, in some experiments performed in this and our other related studies evaluating how allergic mothers influence parameters of allergic disease in offspring, allergen-specific IgG_1 _and IgE responses were virtually absent without profound effects on airway eosinophilia. Since airway eosinophilia can occur in the complete absence of B cells [28], this implies that protection from AAD acquired from allergic mothers in our model has more robust downstream effects on B cell than T cell parameters of disease. Thus, in addition to allergen neutralization, it is likely that maternal antigen-specific IgG_1_/allergen immune complexes contribute to determining the outcome of offspring responses to allergic sensitization [46].

It should be noted that not all maternal Igs have beneficial effects in progeny. Recently, a murine model of peanut allergy demonstrated that maternal derived anti-peanut IgG_1 _is associated with anaphylactic reactions in offspring [47]. In addition, several autoimmune diseases such as systemic lupus erythematosus are known to result in transmission of maternal IgG's that have deleterious effects in progeny [48]. The ability of maternal IgG to mediate differential effects in offspring may be related to affinities for individual FcγRs resulting from Fc glycosylation. IgG is known to contain a single N-linked gycan at Asn^297 ^of the Fc domain, and variations of this covalently linked complex carbohydrate determines binding interactions with individual FcγRs [49,50]. Fc sialyation of IgG_1 _results in a reduced binding affinity for the activating receptor FcγRIII and promotes anti-inflammatory effects through the inhibitory receptor FcγRIIB [49]. It is known that several autoimmune diseases are associated with individual glycoforms of IgG [51,52]. Perhaps the structure of glycans on antigen-specific IgGs varies during the pathogenesis of allergic disease, or is influenced by the environment at the site of the allergen challenge (such as the lung or gut mucosa). Control of post-translational modification of carbohydrate residues on IgGs could be determined during plasma cell differentiation from memory B cells, and/or modified by glycosylation or de-glycosylation enzymes unique to specific tissue environments or disease states. It is possible that physiological changes during lactation itself play a role in the characteristics of maternal IgG acquired by offspring to influence whether maternal IgG transfers increased risk or protection from allergic disease. Recent results from Victor et al. [41] could be supportive of this idea. In their study, neonates nursed by immunized mothers exhibit marked inhibition of B and T cell responses following immunization. In contrast, postnatal injected anti-allergen IgG (purified from serum of immunized mice) failed to modulate expression of FcγRIIB or regulate B or T cell cytokine production.

Our findings suggest a serum concentration limit of absorbed maternal antigen-specific IgG sufficient to protect offspring from AAD. This concept is supported by data from FcRn^+/+ ^or FcRn^+/- ^offspring, where serum levels of antigen-specific IgG_1 _of 10^5 ^- 10^6 ^ng/ml at 52 days of life appeared sufficient to protect offspring from AAD initiated one week later. Significantly reduced levels of antigen-specific IgG_1 _in the serum of weanling FcRn^-/- ^mice, that decayed to negligible levels prior to immunization, resulted in the absence of protection from AAD. Interestingly, the concentration limit of maternal IgG needed to protect offspring from AAD appears to be dependent on the severity of disease elicited in murine models, with lower levels of absorbed allergen-specific IgG_1 _(60-90 ng/ml) being sufficient to protect from mild disease [36]. Additional experimentation aimed at defining the contributions of serum concentrations of maternal IgG, immune complexes and structural glycoforms sufficient to protect offspring from allergic sensitization will be important.

## Conclusion

Our study demonstrates that breast milk factors obtained via FcRn (e.g. IgG) result in reduced severity of allergic airway disease in offspring. Based on these results one could consider increasing maternal antigen-specific IgG levels (e.g. maternal immunization) as a possible method for the prevention of allergic disease in progeny. Further clarification of the IgG levels required to protect offspring, the structural properties of antibodies involved, and their interaction with receptors at various locations (e.g. spleen, intestine, and thymus) in the neonate are important in understanding how passive immunity influences the development of allergy in offspring

## Competing interests

The authors declare that they have no competing interests.

## Authors' contributions

APM supervised the animal experiments, participated in the immunoassays and study design, performed the statistical analysis, and drafted the manuscript. RST reviewed the histopathology and helped to draft the manuscript. ER performed the majority of immunoassays, PCR, and sample collections. EGL helped to direct the animal experiments and immunoassays. LP conceived of the study, participated in its design, coordination, and data analysis; and helped to draft the manuscript. All authors have read and approved the final manuscript.

## References

[B1] Newburg DS Bioactive components of human milk: evolution, efficiency, and protection Adv Exp Med Biol 2001 501 3 10 11787694 10.1007/978-1-4615-1371-1_1

[B2] Labbok MH Clark D Goldman AS Breastfeeding: maintaining an irreplaceable immunological resource Nat Rev Immunol 2004 4 565 572 10.1038/nri1393 15229475

[B3] Gartner LM Morton J Lawrence RA Naylor AJ O'Hare D Schanler RJ Breastfeeding and the use of human milk Pediatrics 2005 115 496 506 10.1542/peds.2004-2491 15687461

[B4] Ip S Chung M Raman G Chew P Magula N Devine D Breastfeeding and maternal and infant health outcomes in developed countries Evid Rep Technol Assess 2007 1 186 PMC4781366 17764214

[B5] Ehrlich P "Collected Papers,"Vol.II Z Hygiene 1892 12 31 44

[B6] Brambell FW Halliday R Brierley J Hemmings WA Transference of passive immunity from mother to young Lancet 1954 266 964 965 13164306 10.1016/s0140-6736(54)91571-8

[B7] Zinkernagel RM Maternal antibodies, childhood infections, and autoimmune diseases N Engl J Med 2001 345 1331 1335 10.1056/NEJMra012493 11794153

[B8] Simister NE Story CM Chen HL Hunt JS An IgG-transporting Fc receptor expressed in the syncytiotrophoblast of human placenta Eur J Immunol 1996 26 1527 1531 10.1002/eji.1830260718 8766556

[B9] Jones EA Waldmann TA The mechanism of intestinal uptake and transcellular transport of IgG in the neonatal rat J Clin Invest 1972 51 2916 2927 10.1172/JCI107116 5080417 PMC292442

[B10] Rodewald R Kraehenbuhl JP Receptor-mediated transport of IgG J Cell Biol 1984 99 159s 164s 10.1083/jcb.99.1.159s 6235233 PMC2275593

[B11] Israel EJ Taylor S Wu Z Mizoguchi E Blumberg RS Bhan A Expression of the neonatal Fc receptor, FcRn, on human intestinal epithelial cells Immunology 1997 92 69 74 10.1046/j.1365-2567.1997.00326.x 9370926 PMC1363983

[B12] Simister NE Mostov KE An Fc receptor structurally related to MHC class I antigens Nature 1989 337 184 187 10.1038/337184a0 2911353

[B13] Roopenian DC Christianson GJ Sproule TJ Brown AC Akilesh S Jung N The MHC class I-like IgG receptor controls perinatal IgG transport, IgG homeostasis, and fate of IgG-Fc-coupled drugs J Immunol 2003 170 3528 3533 12646614 10.4049/jimmunol.170.7.3528

[B14] He W Ladinsky MS Huey-Tubman KE Jensen GJ McIntosh JR Bjorkman PJ FcRn-mediated antibody transport across epithelial cells revealed by electron tomography Nature 2008 455 542 546 10.1038/nature07255 18818657 PMC2773227

[B15] Ghetie V Hubbard JG Kim JK Tsen MF Lee Y Ward ES Abnormally short serum half-lives of IgG in beta 2-microglobulin-deficient mice Eur J Immunol 1996 26 690 696 10.1002/eji.1830260327 8605939

[B16] Chaudhury C Mehnaz S Robinson JM Hayton WL Pearl DK Roopenian DC The major histocompatibility complex-related Fc receptor for IgG (FcRn) binds albumin and prolongs its lifespan J Exp Med 2003 197 315 322 10.1084/jem.20021829 12566415 PMC2193842

[B17] Israel EJ Patel VK Taylor SF Marshak-Rothstein A Simister NE Requirement for a beta 2-microglobulin-associated Fc receptor for acquisition of maternal IgG by fetal and neonatal mice J Immunol 1995 154 6246 6251 7759862

[B18] Akilesh S Christianson GJ Roopenian DC Shaw AS Neonatal FcR expression in bone marrow-derived cells functions to protect serum IgG from catabolism J Immunol 2007 179 4580 4588 17878355 10.4049/jimmunol.179.7.4580

[B19] Montoyo HP Vaccaro C Hafner M Ober RJ Mueller W Ward ES Conditional deletion of the MHC class I-related receptor FcRn reveals the sites of IgG homeostasis in mice Proc Natl Acad Sci USA 2009 106 2788 2793 10.1073/pnas.0810796106 19188594 PMC2650344

[B20] Ghetie V Ward ES Multiple roles for the major histocompatibility complex class I-related receptor FcRn Annu Rev Immunol 2000 18 739 766 10.1146/annurev.immunol.18.1.739 10837074

[B21] Yoshida M Kobayashi K Kuo TT Bry L Glickman JN Claypool SM Neonatal Fc receptor for IgG regulates mucosal immune responses to luminal bacteria J Clin Invest 2006 116 2142 2151 10.1172/JCI27821 16841095 PMC1501111

[B22] Kobayashi K Qiao SW Yoshida M Baker K Lencer WI Blumberg RS An FcRn-dependent role for anti-flagellin immunoglobulin G in pathogenesis of colitis in mice Gastroenterology 2009 137 1746 1756 10.1053/j.gastro.2009.07.059 19664634 PMC2787451

[B23] Jarrett EEE Hall E IgE suppression by maternal IgG Immunology 1983 48 49 58 6848454 PMC1454004

[B24] Victor JR Jr Fusaro AE Duarte AJ Sato MN Preconception maternal immunization to dust mite inhibits the type I hypersensitivity response of offspring J Allergy Clin Immunol 2003 111 269 277 10.1067/mai.2003.39 12589344

[B25] Uthoff H Spenner A Reckelkamm W Ahrens B Wolk G Hackler R Critical role of preconceptional immunization for protective and nonpathological specific immunity in murine neonates J Immunol 2003 171 3485 3492 14500644 10.4049/jimmunol.171.7.3485

[B26] Oshiba A Hamelmann E Takeda K Bradley KL Loader JE Larsen GL Passive transfer of immediate hypersensitivity and airway hyperresponsiveness by allergen-specific immunoglobulin (Ig) E and IgG1 in mice J Clin Invest 1996 97 1398 1408 10.1172/JCI118560 8617871 PMC507198

[B27] von Garnier C Wikstrom ME Zosky G Turner DJ Sly PD Smith M Allergic airways disease develops after an increase in allergen capture and processing in the airway mucosa J Immunol 2007 179 5748 5759 17947647 10.4049/jimmunol.179.9.5748

[B28] Matson AP Thrall RS Rafti E Puddington L Breastmilk from allergic mothers can protect offspring from allergic airway inflammation Breastfeed Med 2009 4 167 174 10.1089/bfm.2008.0130 19301986 PMC2757118

[B29] Matson AP Zhu L Lingenheld EG Schramm CM Clark RB Selander DM Maternal transmission of resistance to development of allergic airway disease J Immunol 2007 179 1282 1291 17617621 10.4049/jimmunol.179.2.1282 PMC3155847

[B30] Wu CA Puddington L Whiteley HE Yiamouyiannis CA Schramm CM Mohammadu F Murine cytomegalovirus infection alters Th1/Th2 cytokine expression, decreases airway eosinophilia, and enhances mucus production in allergic airway disease J Immunol 2001 167 2798 2807 11509625 10.4049/jimmunol.167.5.2798

[B31] Lohning M Stroehmann A Coyle AJ Grogan JL Lin S Gutierrez-Ramos JC T1/ST2 is preferentially expressed on murine Th2 cells, independent of interleukin 4, interleukin 5, and interleukin 10, and important for Th2 effector function Proc Natl Acad Sci USA 1998 95 6930 6935 10.1073/pnas.95.12.6930 9618516 PMC22690

[B32] Masopust D Vezys V Marzo AL Lefrançois L Preferential localization of effector memory cells in nonlymphoid tissue Science 2001 291 2413 2417 10.1126/science.1058867 11264538

[B33] Seymour BWP Gershwin LJ Coffman RL Aerosol-induced immunoglobulin (Ig)-E unresponsiveness to ovalbumin does not require CD8^+ ^or T cell receptor (TCR)-γ/δ^+ ^T cells or interferon (IFN)-γ in a murine model of allergen sensitization J Exp Med 1998 187 721 731 10.1084/jem.187.5.721 9480982 PMC2212168

[B34] Roopenian DC Christianson GJ Sproule TJ Human FcRn transgenic mice for pharmacokinetic evaluation of therapeutic antibodies Methods Mol Biol 2010 602 93 104 full_text 20012394 10.1007/978-1-60761-058-8_6

[B35] Schramm CM Puddington L Yiamouyiannis CA Lingenheld EG Whiteley HE Wolyniec WW Proinflammatory roles of TCRγδ and TCRαβ lymphocytes in a murine model of asthma Am J Respir Cell Mol Biol 2000 22 218 225 10657943 10.1165/ajrcmb.22.2.3620

[B36] Nakata K Kobayashi K Ishikawa Y Yamamoto M Funada Y Kotani Y The transfer of maternal antigen-specific IgG regulates the development of allergic airway inflammation early in life in an FcRn-dependent manner Biochem Biophys Res Commun 2010 395 238 243 10.1016/j.bbrc.2010.03.170 20362552 PMC3990410

[B37] Friedman NJ Zeiger RS The role of breast-feeding in the development of allergies and asthma J Allergy Clin Immunol 2005 115 1238 1248 10.1016/j.jaci.2005.01.069 15940141

[B38] Polte T Hennig C Hansen G Allergy prevention starts before conception: maternofetal transfer of tolerance protects against the development of asthma J Allergy Clin Immunol 2008 122 1022 1030 10.1016/j.jaci.2008.09.014 19000583

[B39] Fusaro AE Maciel M Victor JR Oliveira CR Duarte AJ Sato MN Influence of maternal murine immunization with Dermatophagoides pteronyssinus extract on the type I hypersensitivity response in offspring Int Arch Allergy Immunol 2002 127 208 216 10.1159/000053865 11979046

[B40] Jarrett E Hall E Selective suppression of IgE antibody responsiveness by maternal influence Nature 1979 280 145 147 10.1038/280145a0 95350

[B41] Victor JR Muniz BP Fusaro AE de Brito CA Taniguchi EF Duarte AJ Maternal immunization with ovalbumin prevents neonatal allergy development and up-regulates inhibitory receptor Fc gamma RIIB expression on B cells BMC Immunol 2010 11 11 10.1186/1471-2172-11-11 20222978 PMC2848204

[B42] Leme AS Hubeau C Xiang Y Goldman A Hamada K Suzaki Y Role of breast milk in a mouse model of maternal transmission of asthma susceptibility J Immunol 2006 176 762 769 16393959 10.4049/jimmunol.176.2.762

[B43] Siegrist CA Neonatal and early life vaccinology Vaccine 2001 19 3331 3346 10.1016/S0264-410X(01)00028-7 11348697

[B44] Albrecht P Ennis FA Saltzman EJ Krugman S Persistence of maternal antibody in infants beyond 12 months: mechanism of measles vaccine failure J Pediatr 1977 91 715 718 10.1016/S0022-3476(77)81021-4 909009

[B45] Lambert PH Liu M Siegrist CA Can successful vaccines teach us how to induce efficient protective immune responses? Nat Med 2005 11 S54 S62 10.1038/nm1216 15812491

[B46] Mosconi E Rekima A Seitz-Polski B Kanda A Fleury S Tissandie E Breast milk immune complexes are potent inducers of oral tolerance in neonates and prevent asthma development Mucosal Immunol 2010 3 doi:10.1038/mi.2010.23 10.1038/mi.2010.23 20485331

[B47] Lopez-Exposito I Song Y Jarvinen KM Srivastava K Li XM Maternal peanut exposure during pregnancy and lactation reduces peanut allergy risk in offspring J Allergy Clin Immunol 2009 124 1039 1046 10.1016/j.jaci.2009.08.024 19895992 PMC2801422

[B48] Tincani A Rebaioli CB Frassi M Taglietti M Gorla R Cavazzana I Pregnancy and autoimmunity: maternal treatment and maternal disease influence on pregnancy outcome Autoimmun Rev 2005 4 423 428 10.1016/j.autrev.2005.03.001 16137607

[B49] Kaneko Y Nimmerjahn F Ravetch JV Anti-inflammatory activity of immunoglobulin G resulting from Fc sialylation Science 2006 313 670 673 10.1126/science.1129594 16888140

[B50] Nimmerjahn F Ravetch JV Divergent immunoglobulin G subclass activity through selective Fc receptor binding Science 2005 310 1510 1512 10.1126/science.1118948 16322460

[B51] Parekh RB Dwek RA Sutton BJ Fernandes DL Leung A Stanworth D Association of rheumatoid arthritis and primary osteoarthritis with changes in the glycosylation pattern of total serum IgG Nature 1985 316 452 457 10.1038/316452a0 3927174

[B52] Holland M Yagi H Takahashi N Kato K Savage CO Goodall DM Differential glycosylation of polyclonal IgG, IgG-Fc and IgG-Fab isolated from the sera of patients with ANCA-associated systemic vasculitis Biochim Biophys Acta 2006 1760 669 677 16413679 10.1016/j.bbagen.2005.11.021

